# Chronic constipation and the brain-gut-microbiome axis: the role of 5-HT signaling and Traditional Chinese Medicine in pathophysiology and treatment

**DOI:** 10.3389/fmed.2025.1706411

**Published:** 2026-01-23

**Authors:** Shuangshuang Zhang, Xiao Song, Yan Wen, Guosheng Wang

**Affiliations:** 1 Department of Traditional Chinese Medicine, Shandong Provincial Second People’s Hospital, Jinan, Shandong, China; 2 Department of Rehabilitation Hospital, Shandong Provincial Second People’s Hospital, Jinan, Shandong, China

**Keywords:** 5-hydroxytryptamine, brain-gut-microbiome axis, chronic constipation, signaling pathway, Traditional Chinese Medicine

## Abstract

Chronic constipation (CC) is a prevalent functional gastrointestinal disorder involving complex interactions among the brain-gut-microbiome axis, with 5-hydroxytryptamine (5-HT) as a key signaling node. Aberrations in 5-HT synthesis, release, receptor expression, or reuptake disrupt gastrointestinal motility, contributing to CC pathogenesis. Traditional Chinese Medicine (TCM), including herbal compounds, monomers, acupuncture, and tuina, exerts therapeutic effects by modulating the 5-HT signaling pathway. Animal studies demonstrate that TCM interventions regulate gut microbiota, promote 5-HT production via metabolites like short-chain fatty acids (SCFAs) and bile acids, and target receptors (e.g., 5-HT_3_R, 5-HT_4_R) to enhance intestinal motility. Clinical trials validate TCM’s efficacy in normalizing 5-HT levels and improving symptoms, with advantages in safety and holistic regulation. However, important gaps remain, including incomplete understanding of upstream and downstream 5-HT signaling mechanisms, paradoxical 5-HT expression, and limited investigation of comorbid emotional disorders. Future studies should explore how TCM interventions modulate the gut microbiota–5-HT axis and inflammation-related pathways to provide novel insights into CC management.

## Introduction

Chronic constipation (CC) is a prevalent and frequently occurring functional gastrointestinal disorder characterized by difficult defecation, reduced defecation frequency, and hard/stool consistency (1). The global prevalence of CC is approximately 15%, with a higher incidence in older populations and females (2). Etiologically, CC is categorized into functional, organic, and drug-induced subtypes, further divided into primary (functional) and secondary constipation. Pathophysiologically, it is classified into normal transit constipation, slow transit constipation (STC), dyssynergic defecation, and mixed constipation (3).

The Rome IV criteria attribute functional gastrointestinal disorders to abnormal brain-gut interactions. The pathogenesis of constipation is intricately linked to intestinal flora imbalance, enteric nervous system (ENS) dysfunction, central nervous system (CNS) abnormalities, and dysregulation of brain-gut neurotransmitters, forming a reciprocal and vicious cycle (4). The brain-gut-microbiome axis has emerged as a key concept in gastrointestinal research, with serotonin (5-hydroxytryptamine, 5-HT) identified as a critical nodal molecule bridging this axis (5, 6). As a bidirectional regulator, 5-HT functions as a neurotransmitter, hormone, and growth factor, participating in the regulation of mood, intestinal mucosal growth, gastrointestinal motility, secretion, and enteric neuron maturation (7).

Mounting evidence indicates that aberrations in the 5-HT signaling pathway–encompassing synthesis, release, receptor expression, or reuptake–are closely associated with the development and progression of constipation (8). Thus, modulating the 5-HT signaling pathway represents a potential therapeutic target for CC.

With changing dietary patterns, lifestyle rhythms, and psychosocial factors, the prevalence of CC is on the rise. Complications include hemorrhoids, anal fissures, and comorbid psychiatric disorders, with severe cases potentially increasing cancer risk, significantly impairing patients’ quality of life and daily functioning (9, 10). Current clinical management relies on laxatives and 5-HT4 receptor agonists to promote gastrointestinal motility (11); however, long-term use often leads to adverse events such as drug dependence and gastric injury (12). There is an urgent need for effective treatments with minimal side effects.

Traditional Chinese Medicine (TCM), characterized by safety, low toxicity, and affordability, offers unique advantages in treating constipation through oral herbal formulations, external applications, acupuncture, cupping, and tuina (tui-na) massage (13, 14). Recent research highlights the 5-HT signaling pathway as a focal point in understanding constipation pathophysiology. This review synthesizes advances in TCM-mediated regulation of the 5-HT signaling pathway for CC, aiming to inform future clinical and basic research.

## 5 -HT signaling pathway, constipation, and the brain-gut-microbiome axis

### Synthesis, release, receptor expression, and reuptake of 5-HT

Approximately 5% of 5-HT is sourced from the CNS, with the remaining 95% originating from the gastrointestinal tract. Among this, 90% is synthesized by enterochromaffin cells (ECs), and the rest is produced by the myenteric plexus of the ENS (4). Tryptophan, the precursor for 5-HT synthesis, is catalyzed by tryptophan hydroxylase (TPH) to form 5-hydroxytryptophan, which is then decarboxylated by aromatic L-amino acid decarboxylase to generate 5-HT. TPH1 and TPH2 are rate-limiting enzymes in 5-HT synthesis; TPH1, predominantly expressed in ECs, is responsible for peripheral 5-HT production, while TPH2 is mainly expressed in enteric neurons and central 5-HTergic neurons (15).

Once 5-HT binds to its receptors and exerts its function, it rapidly dissociates and is reuptaken by the serotonin transporter (SERT), thus terminating the signal and preventing receptor overstimulation and desensitization (16). To date, seven types of 5-HT receptors (5-HTRs) have been identified, including 5-HTA,B,D,E,ER, 5-HT2A,B,cR, 5-HT_3_R, 5-HT_4_R, 5-HTsA,BR, 5-HT6R and 5-HT7R^1^8. Among them, 5-HT_3_R and 5-HT_4_R play significant roles in regulating gastrointestinal functions and have been extensively studied in the context of constipation. 5-HT_3_R, the only ligand-gated ion channel receptor among 5-HTRs, enhances the activity of interstitial cells of Cajal by modulating extracellular Ca^2+^ concentration and increases neurotransmitter secretion, thereby improving intestinal motility (17). 5-HT2R, aG protein-coupled receptor, is the most abundant 5-HTR exposed to the intestinal lumen. It can induce the release of acetylcholine (Ach), substance P (SP), and calcitonin gene-related peptide, coordinating the contraction and relaxation of intestinal smooth muscle and regulating digestive juice secretion (18).

**FIGURE 1:**
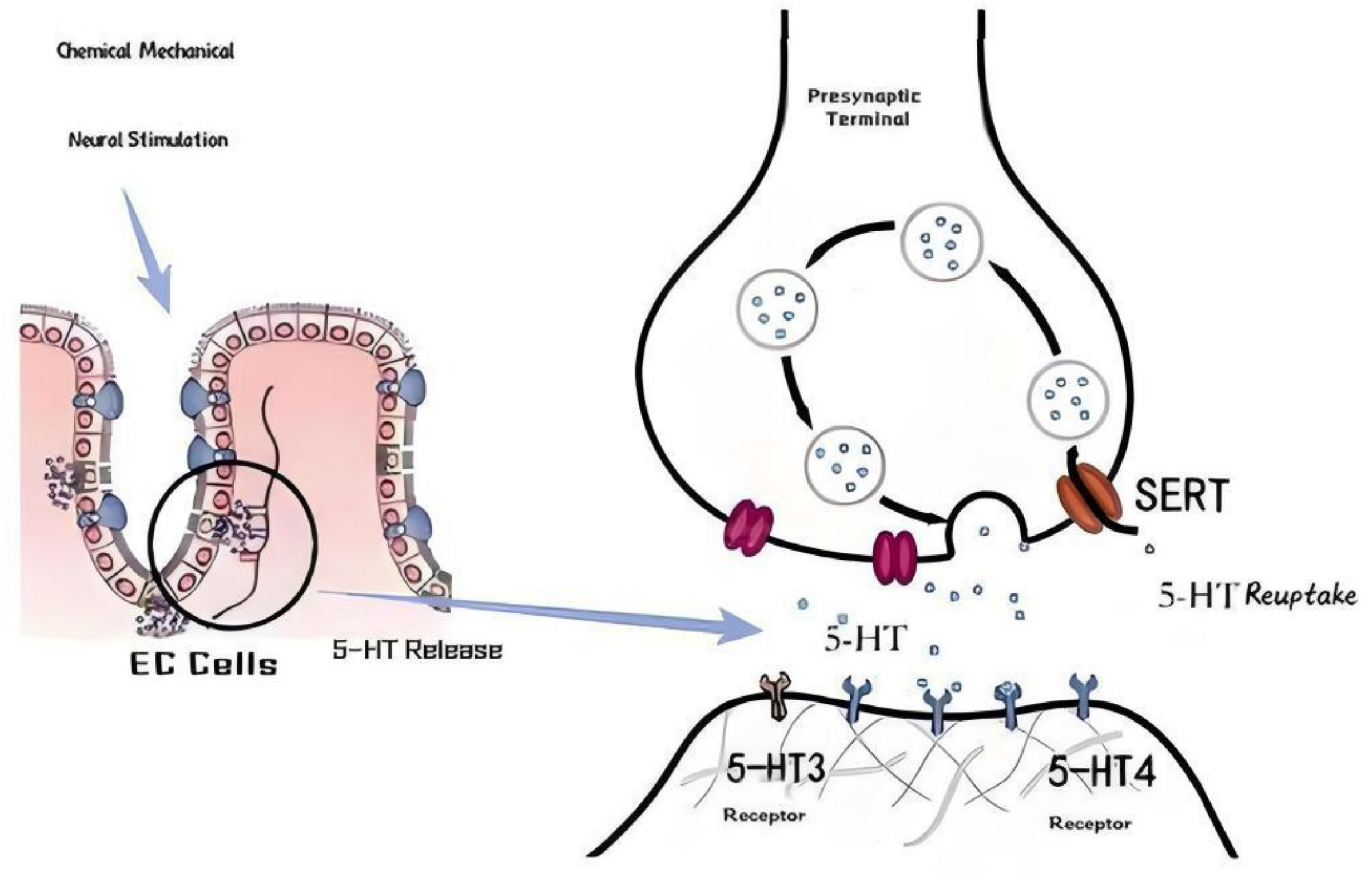
Mechanism of action and reuptake of 5-HT in the intestine.

Enterochromaffin cells (EC) are distributed on the top of the intestinal villi. They can sense the contents of the intestine and convert the signals in the intestinal cavity into biochemical signals 70, so they are called “taste buds of the intestine.” Under the activation of nerve stimulation, intestinal cavity/mucosal stimulation (including physical and chemical stimulation such as expansion, food, acid, hypertonic/hypotonic solution), EC cells release 5-HT to the extracellular space through vesicles (19). Its secretion amount is much greater than that of central or peripheral serotonergic neurons. The 5-HT secreted by it not only permeates the mucosa, but also reaches the gastrointestinal cavity and can be absorbed into the blood. It is taken up and concentrated by platelets in the blood and is the only source of 5-HT for platelets (20). The 5-HT secreted on the intestinal surface of EC cells can stimulate exogenous and endogenous primary afferent nerves (IPANs), which are located in the submucosal and intermuscular nerve plexuses. Submucosal IPANs are related to intestinal peristalsis and secretory reflexes, while myenteric plexus IPANs are related to large migration contractions. Submucosal IPANs are activated by 5-HT1p receptors, and myenteric plexus IPANs are activated by 5-HT3 receptors (21). Both submucosal and myenteric plexus IPANs are cholinergic (Ach) nerves, and the former can also release calcitonin gene-related peptide (CGRP) (22). The 5-HT4 receptors located before the synapse can increase the release of Ach and CGRP by IPANs, thereby enhancing the strength of cholinergic nerve conduction and increasing the release of CGRP to promote the diffusion of conduction along the intestinal wall (23). As a result, subthreshold stimulation will achieve the effect of activating the reflex. After exerting its effect, 5-HT is taken into intestinal cells through the mediation of 5-hydroxytryptamine reuptake transporter (SERT) for inactivation (24). See Figure 1.

## Aberrations in the 5-HT signaling pathway leading to constipation

The 5-HT signaling pathway is involved in regulating gastrointestinal motility, sensation, and secretion. Abnormalities in any step of 5-HT synthesis, release, receptor expression, or reuptake can disrupt gastrointestinal function and lead to constipation. The potential mechanisms are as follows:

① Altered 5-HT Levels: studies have shown that 5-HT content in CC patients and constipation models is lower than that in non-constipated individuals (25–27). However, experiments also detected increased 5-HT expression in the colons of constipated rats (28, 29). Additionally, in CC patients without reduced SERT, the 5-HT content in colon tissue is elevated (30). Therefore, the former case of constipation may be due to decreased 5-HT, which reduces gastrointestinal peristalsis and secretory reflexes. In the latter case, increased 5-HT affects enteric nerve conduction, keeping the colon in a continuously contracted state, and the increased availability of 5-HT causes receptor desensitization.
② Imbalanced Receptor Expression: multiple studies have reported decreased expression of 5-HT_3_R and 5-HT_4_R in the colonic mucosa of STC patients and rats. This reduction affects the transmission of intestinal sensation to the central nervous system, leading to a decreased urge to defecate (31–35). 5-HT_4_R can regulate colonic smooth muscle relaxation, and both constipated rats and patients exhibit increased 5-HT2R content, suggesting that the high expression of this receptor may inhibit normal intestinal peristalsis and secretory reflexes (36).
③ Reduced SERT Expression: SERT expression is significantly decreased in the mucosal layer and myenteric plexus of STC patients (37). CHEN et al. demonstrated that SERT - deficient mice experience irregular alternation of diarrhea and constipation. Diarrhea may be attributed to enhanced 5-HT signaling, while constipation may result from increased 5-HT release leading to receptor desensitization (38).

## 5 -HT and the brain - gut - microbiome axis

### 5 -HT as a key node in the brain - gut - microbiome axis

The brain - gut - microbiome axis is a bidirectional communication network between the gut and the brain, composed of gut microbiota and their metabolites, the ENS, CNS, sympathetic and parasympathetic nerve branches, the neuroimmune system, and the neuroendocrine system (39). 5-HT is a crucial regulator of ENS and CNS development and function. During CNS development, 5-HT has neurogenic effects, regulating cell division, migration, and differentiation, and later participates in regulating mood (such as depression and anxiety) and cognition. In the peripheral nervous system, 5-HT is essential for ENS development, intestinal motility, permeability, and the growth and differentiation of intestinal epithelial cells (40).

The gut microbiota communicates directly with the CNS via the vagus nerve or through the production of bioactive metabolites that directly affect gut function. Some metabolites can cross the blood - brain barrier and indirectly act on the CNS (41). The gut microbiota and their metabolites can regulate 5-HT production, and host 5-HT metabolism influences microbial composition and function (42). Given the interactions between serotonin and the CNS, ENS, and microbiome, 5-HT may serve as a common node in the brain - gut - microbiome axis.

## Regulation of 5-HT synthesis by gut microbiota and their metabolites

The regulation of 5-HT by gut microbiota and their metabolites may be a crucial link in influencing intestinal motility. In germ - free mice, the expression of TPH1 in the colon is reduced, and microbiota transplantation can increase its expression to promote 5-HT biosynthesis (43). In an experiment involving the colonization of a 5-HT - producing genetically engineered probiotic strain (EeN - 5-HT), after 2-weeks gavage of EeN - 5-HT, 5-HT levels increased in constipated mice, and intestinal motility was significantly improved (2). Moreover, numerous studies indicate that the gut microbiota, especially spore - forming bacteria, can stimulate 5-HT production in ECs through TPH1 (44).

The most studied metabolites affecting 5-HT synthesis are short - chain fatty acids (SCFAs) and secondary bile acids, particularly deoxycholic acid (45, 46). SCFAs, such as acetic acid, propionic acid, and butyric acid, are produced by anaerobic fermentation in the gut by the two dominant phyla in the human gut microbiota, Firmicutes and Bacteroidetes (47). SCFAs can stimulate free fatty acid receptors in ECs, increasing TPH1 expression and 5-HT production. Bile acids (BA) can activate ECs and the G protein - coupled bile acid receptor 5 (TGR5), promoting 5-HT secretion (48). Recent experiments have revealed that microbial metabolites, such as α - tocopherol, cholic acid, tyramine, and p - aminobenzoic acid, can also increase 5-HT secretion in ECs (49). Other studies have shown that bacterial toxins, such as cholera toxin and Escherichia coli lipopolysaccharide, have the same effect (50). Tryptamine produced by microorganisms can also induce 5-HT synthesis in myenteric neurons.

Recently, it has been found that even when all endogenous 5-HT in the gut has been genetically or pharmacologically (51) cleared, 5-HT antagonists still have the same or stronger inhibitory effects on gastrointestinal motility, while exogenous 5-HT can strongly increase gastrointestinal transit function in many tested species (52). Therefore, 5-HT derived from gut microbiota may provide a potential therapeutic strategy for constipation (42). In particular, the holistic diagnosis and treatment approach of Traditional Chinese Medicine, which comprehensively regulates and maintains gut homeostasis, holds great promise.

## Regulation of 5-HT signaling pathway by Traditional Chinese Medicine in the treatment of constipation

In Traditional Chinese Medicine (TCM), constipation is primarily associated with dysfunction of the large intestine, while also involving imbalances in the lung, spleen (stomach), liver, and kidney. Pathological factors such as yangming dryness-heat consuming body fluids, qi stagnation impairing intestinal conduction, cold pathogenic factors congealing in the intestines, qi deficiency failing to propel contents, blood deficiency depriving the intestines of nourishment, yin deficiency causing intestinal dryness, and yang deficiency weakening intestinal warmth can all lead to impaired downward conduction of the large intestine, resulting in constipation. The core therapeutic principle is to restore the conduction function of the intestinal fu-organ. TCM offers diverse treatment modalities, including oral herbal decoctions, external applications, acupuncture, cupping, and tuina (tui-na) massage. Through syndrome differentiation and holistic regulation of the brain-gut-microbiome axis, TCM interventions target the 5-HT signaling pathway with multi-component, multi-effect, and multi-target actions, effectively alleviating constipation symptoms and improving quality of life, thus demonstrating distinct advantages.

## Animal experimental studies

### Chinese herbal compounds

Traditional Chinese Medicine emphasizes syndrome differentiation and holistic approaches, where multi-herb formulations (compounds) maximize therapeutic efficacy by synergistic effects (53). Animal studies on TCM compounds for constipation can be categorized by their functions: qi-supplementing and yin-nourishing, yang-warming, qi-regulating, and heat-clearing and intestine-moistening.

Qi-supplementing and yin-nourishing compounds: Shouhui Tongbian Capsule, an approved TCM patent drug for constipation since 2015, possesses the effects of nourishing yin, supplementing qi, eliminating turbidity, and promoting defecation (54). In studies using loperamide hydrochloride (LH)-induced constipated mice, Shouhui Tongbian Capsule was found to correct intestinal flora dysbiosis, activate bacterial metabolite-mediated intestinal 5-HT synthesis, and protect enteric neuron differentiation, thereby enhancing intestinal motility (53). To verify whether its effect on 5-HT synthesis depends on regulating intestinal flora homeostasis, fecal transplantation from drug-treated mice to untreated constipated mice resulted in increased colonic TPH1 mRNA expression, confirming the central role of intestinal flora in its therapeutic effect (55). Although Yangyin Yiqi Runchang Formula (55) and Maren Pills (56) also improve intestinal flora imbalance and increase 5-HT levels, their reliance on gut microbiota and metabolites to mediate 5-HT-related laxative effects remains unconfirmed due to the lack of germ-free models or fecal transplantation experiments (57).

Heat-clearing and intestine-moistening compounds: Maren Pills, widely used clinically for constipation, regulate the relative abundance of *Lactobacillus* and *Clostridium* to improve colonic flora structure in STC rats. They also promote the secretion of short-chain fatty acids (SCFAs, e.g., acetate, propionate, butyrate) and upregulate colonic 5-HT and 5-HT_4_R expression, thereby activating the 5-HT pathway to enhance intestinal peristalsis (58).

Yang-warming compounds: a study by Zhang Yuanzhe et al. (58) demonstrated that the compatibility of Aconiti Lateralis Radix Praeparata and Cinnamomi Cortex upregulates colonic 5-HT_3_R/5-HT_4_R expression, 5-HT content, and chromogranin A (CgA) levels in STC rats. CgA, an acidic soluble protein in chromaffin granules, serves as a marker for chromaffin cells and is distributed in nerve terminals, peripheral nervous system, CNS, and intestinal endocrine tissues, contributing to maintaining gastrointestinal tone and motility (59). Additionally, Jiang Lingfang et al. (60) reported that Jichuan Decoction ameliorates constipation symptoms in STC rats induced by compound diphenoxylate (CDT) gavage, possibly by increasing TPH1 expression and 5-HT levels.

Qi-regulating compounds: the compatibility of Aurantii Fructus Immaturus and Atractylodis Macrocephalae Rhizoma (61), as well as Simotang (62), upregulate the mRNA and protein expression of colonic 5-HT_3_R and 5-HT_4_R in STC rats, thereby promoting intestinal motility (Table 1).

**TABLE 1:** Mechanisms of TCM compounds in treating constipation: focus on gut microbiota and 5-HT pathway.

Category	Compound name	Study model	Core mechanism
Qi-supplementing and yin-nourishing compounds	Shouhui Tongbian Capsule	Loperamide-induced constipated mice	FMT-confirmed: modulates flora to increase 5-HT production and protect neurons
	Yangyin Yiqi Runchang Formula and Maren Pills	Model not specified	Increases 5-HT; microbiota role unconfirmed
Heat-clearing and intestine-moistening compounds	Maren Pills	STC rats	Improves flora structure and SCFAs to activate 5-HT pathway
Yang-warming	Aconite and Cinnamon	STC rats	Upregulates 5-HT, its receptors, and CgA to enhance motility
	Jichuan Decoction	Compound diphenoxylate-induced STC rats	Increases TPH1 expression and 5-HT levels
Qi-regulating	Aurantii, Atract and Simotang	STC rats	Promotes intestinal motility by targeting 5-HT receptors

In summary, Chinese herbal compounds exert therapeutic effects on constipation primarily through the “intestinal flora-5-HT pathway-intestinal motility axis.” Animal studies can verify whether herbal-induced improvements in 5-HT synthesis and gastrointestinal motility depend on altered gut microbiota using germ-free models and fecal transplantation. However, further research is needed to identify specific gut microbial species, their metabolites, and active herbal components that mediate these effects.

## Herbal monomers

With advances in TCM pharmacology, herbal monomers–characterized by clear components and significant efficacy–have shown great potential in regulating the 5-HT signaling pathway to alleviate constipation.

Hao et al. (63) investigated the mechanism of *Platycodon grandiflorum* polysaccharides in improving intestinal motility disorders in constipated rats. 16S rRNA sequencing revealed that, compared to the model group, the polysaccharide-treated group had lower Firmicutes and higher Bacteroidetes abundance, with dose-dependent changes in gut microbiota composition. Additionally, the polysaccharides significantly increased 5-HT secretion and the expression of related proteins [e.g., TPH1, 5-HT_4_R, and transient receptor potential ankyrin 1 (TRPA1)]. TRPA1, a non-selective cation channel expressed in small intestinal ECs and colonic mesenchymal cells, senses luminal stimuli and mediates 5-HT release from ECs to regulate gastrointestinal motility (64).

*Amomum tsaoko*, known for regulating gastrointestinal function, anti-inflammation, and anti-tumor effects (65), is used for syndrome such as cold-damp obstruction in the spleen and stomach, abdominal distension, diarrhea, and vomiting. Hu et al. (66) explored the laxative mechanism of total flavonoids from *A. tsaoko* in STC mice, showing that they modify gut microbial structure by increasing beneficial bacteria (e.g., *Lactobacillus*, *Bacillus*) and reducing dominant symbionts (e.g., Lachnospiraceae), thereby maintaining intestinal homeostasis. Concurrently, these flavonoids upregulate serum 5-HT levels and the mRNA expression of 5-HT2AR, TRPA1, phospholipase A2 (PLA2), and cyclooxygenase-2 (COX2), which are involved in 5-HTergic synaptic pathways.

Atractylenolide I, an active component of Atractylodis Macrocephalae Rhizoma (a spleen-invigorating herb), positively regulates gut microbiota diversity in constipated rats. Its mechanism may involve increasing the proportion of *Bacteroides* and *Parabacteroides*, promoting propionate release, and enhancing 5-HT expression to alleviate constipation (67). TGR5, a bile acid membrane receptor widely distributed in organs including the spleen, lung, liver, kidney, gastrointestinal tract, and bone marrow, acts as a metabolic regulator in bile acid metabolism, energy homeostasis, and gastrointestinal motility regulation; TRPA1 is a downstream regulator of TGR5 (68). Studies have found that paeoniflorin activates TGR5/TRPA1 signaling to induce 5-HT release from ECs and TPH1 expression, thereby improving constipation (69). Hesperidin, a flavonoid glycoside and major component of Aurantii Fructus, enhances gastrointestinal transit in STC rats by increasing 5-HT_4_R expression and intracellular free Ca^2+^ levels (70).

These findings indicate that TGR5, TRPA1, COX2, and PLA2 participate in 5-HT signaling as supplementary factors, holding significance for future research on constipation and TCM interventions. Preclinical studies on herbal monomers provide a theoretical basis for developing potential laxative drugs, with substantial prospects for novel drug development.

## Acupuncture and tuina

Acupuncture and tuina demonstrate definite efficacy and high safety in treating constipation, but their mechanisms remain incompletely understood. Thus, exploring their effects using modern molecular techniques is crucial to support clinical application.

Li Shuo et al. (71) found that acupuncture at Houhai (Changqiang), Housanli (Zusanli), and Dachangshu exerted distinct therapeutic pathways in constipated rats: Changqiang significantly reduced serum 5-HT and somatostatin (SS) levels while upregulating colonic 5-HT_3_R/5-HT_4_R mRNA expression; Zusanli showed similar effects but did not reduce SS; Dachangshu increased serum vasoactive intestinal peptide (VIP) and substance P (SP) levels and upregulated 5-HT_4_R mRNA expression. Additionally, Wang Haiyan et al. (72) compared three acupuncture techniques (filiform needle, acupoint injection, and catgut embedding) at Changqiang, finding that all regulated VIP, SP, and 5-HT pathways, with efficacy comparable to mosapride, and catgut embedding and acupoint injection superior to filiform needle.

Xu Mingmin (73) explored the molecular mechanism of electroacupuncture at He-Mu points (Tianshu, Shangjuxu) in regulating gut microbiota for constipation. Results suggested that acupuncture may upregulate Staphylococcaceae, promote butyrate production, and stimulate 5-HT and 5-HT_4_R expression to enhance gastrointestinal transit. Notably, in microbiota-depleted mice, acupuncture’s regulatory effects on transit, SCFAs, 5-HT, and 5-HT_4_R were inhibited, confirming that gut microbiota modulation is critical to its efficacy. Ma Jiaze et al. (74) confirmed that electroacupuncture at Zhongliao and Xialiao promotes intestinal motility by targeting multiple nodes of the 5-HT signaling system, possibly via improving gut microbiota structure and increasing fecal butyrate and acetate levels–though specific bacteria influencing SCFAs require further investigation.

Other experiments showed that electroacupuncture at “Baihui” and/or “Zusanli” increased colonic TPH and 5-HT expression in constipated rats (74), while combined stimulation of Tianshu and Zusanli upregulated SERT and 5-HT_3_R levels (75). Wang Dongliang et al. (76) reported that abdominal tuina (pressing and kneading) at Guanyuan and Zhongwan acupoints elevated colonic 5-HT_3_R and 5-HT_4_R levels in STC rats, improving disease status and outcomes.

In summary, acupuncture and tuina also regulate the “gut microbiota-5-HT pathway-intestinal motility axis” to alleviate constipation. Key acupoints include back-shu points, front-mu points, and lower-he points of the large intestine. Differences in molecular expression exist between acupoints and techniques, highlighting the need for comparative studies to clarify 5-HT-related mechanisms and develop individualized protocols for optimal efficacy.

## Clinical studies

Animal experimental findings cannot be directly extrapolated to humans, necessitating rigorous clinical trials. Clinical studies primarily modulate 5-HT signaling, promote intestinal motility, and improve constipation through oral TCM, acupuncture, or TCM-WM combination therapy. Herbal formulas align with animal studies, focusing on qi-supplementing and yin-nourishing, spleen-kidney warming, and qi-regulating and intestinal-moistening strategies. Notably, clinicians often overlook the psychological aspects of chronic constipation and their bidirectional relationship with the condition. Thus, TCM interventions based on the brain-gut-microbiome axis, leveraging holistic concepts, show distinct advantages.

Yang Chenting (77) observed the clinical efficacy of LiuMo Decoction combined with SiNi Powder in treating qi-stagnation functional constipation with anxiety, finding that the herbal formula corrected 5-HT secretion disorders, significantly improved constipation and anxiety, and showed high safety with no adverse effects, warranting clinical promotion. Shi Zhe et al. (78) randomized 160 drug-dependent constipation patients with qi-yin deficiency to receive modified Qibang Formula (observation group) or lactulose (control group) for 8 weeks. The modified formula showed superior short- and medium-term efficacy, with higher 5-HT levels in the observation group; 5-HT_4_R levels increased post-treatment but did not differ from the control, suggesting potential involvement of other 5-HTR subtypes requiring further study.

Niu Mingliao et al. (79) treated elderly STC patients with spleen-kidney yang deficiency using modified Jichuan Decoction, with the control group receiving Biantong Capsules. After 4 weeks, 5-HT and 5-HT_4_R levels were significantly higher in the observation group, indicating potential differences in 5-HT_4_R regulation between qi-yin supplementation and spleen-kidney warming.

Hu Minjie (80) investigated the effects of modified Huangqi Decoction on 5-HT, gut microbiota, and SCFAs in patients with qi-deficiency constipation. The study group received the decoction plus mosapride, while the control group received quadruple viable bifidobacteria plus mosapride for 2 weeks. Both groups showed increased serum 5-HT, improved gut dysbiosis (without significant differences in phylum/genus levels), and decreased SCFAs. Possible reasons included small sample size (23 cases), short duration, lack of dose stratification, and subjective biases in fecal collection.

Xu Huafang et al. (81) used electroacupuncture at Tianshu, Shangjuxu, and Fujie in functional constipation patients, with the control group receiving sham electroacupuncture. After 8 weeks, the observation group showed better efficacy and higher 5-HT levels. Wei Xiaoli et al. (82) randomized 100 patients with habitual constipation to receive mosapride alone (control) or combined with acupuncture at Tianshu; after 2 weeks, the combined group showed lower 5-HT levels and superior symptom improvement (Table 2).

**TABLE 2:** Clinical efficacy of TCM therapies for constipation: modulation of 5-HT and gut microbiome.

Intervention	Patient population	Key findings on 5-HT pathway	Clinical outcome
LiuMo Dct + SiNi powder	FC with anxiety	Corrected 5-HT secretion disorders	Improved constipation and anxiety; high safety
Modified Qibang	Formula drug-dep. FC (Qi-Yin Def)	Increased 5-HT and 5-HT_4_R (vs. baseline)	Superior short and medium-term efficacy vs. lactulose
Modified Jichuan Dct	Elderly STC (Spleen-Kidney Yang Def)	Increased 5-HT and 5-HT_4_R levels vs. control	Better efficacy than Biantong Capsules
Modified Huangqi Dct + mosapride	Qi-deficiency FC	Increased serum 5-HT; improved gut dysbiosis	Similar efficacy to probiotics + mosapride
Electroacupuncture (Tianshu, etc.)	Functional constipation	Increased 5-HT levels vs. sham group	Better efficacy after 8 weeks
Acupuncture + mosapride	Habitual constipation	Decreased 5-HT levels; superior symptoms	Better efficacy than mosapride alone after 2 weeks

In conclusion, TCM treatments exert holistic, benign regulation of 5-HT signaling, normalizing 5-HT or 5-HTR levels to alleviate constipation. Mechanistic differences between therapies and formulas require further exploration. Given the challenges in conducting rigorous clinical trials, optimizing methodologies and conducting multi-center, large-sample studies are essential.

## Discussion

Chronic constipation (CC) is a prevalent clinical symptom with suboptimal therapeutic outcomes, and research on TCM-based interventions for constipation remains dynamically evolving (83). This review synthesizes the associations between constipation, the 5-HT signaling pathway, and the brain-gut-microbiome axis, alongside advances in TCM-mediated therapies. Animal studies have elucidated the specific mechanisms by which TCM–encompassing herbal compounds, monomers, acupuncture, and tuina–modulates the 5-HT signaling pathway to alleviate constipation. Clinical trials, focusing on herbal formulas, acupuncture, and TCM-western medicine combinations, have validated therapeutic efficacy by measuring changes in serum 5-HT and 5-HT receptor (5-HTR) levels before and after treatment. Collectively, TCM exerts specific regulatory effects on 5-HT signaling, with the brain-gut-microbiome axis as a critical pathway, highlighting its potential as a novel target for constipation prevention and treatment with broad application prospects.

Despite these insights, several challenges and unresolved questions persist:

The classical 5-HT signaling pathway, traditionally defined to include enterochromaffin cells (ECs), tryptophan hydroxylase (TPH), 5-HT, 5-HTRs, and serotonin transporter (SERT), lacks integration of upstream and downstream molecules. Based on current evidence, supplementary components should include gut microbiota, microbial metabolites [e.g., short-chain fatty acids (SCFAs), bile acids (BAs)], chromogranin A (CgA), TGR5, transient receptor potential ankyrin 1 (TRPA1), cyclooxygenase-2 (COX2), and phospholipase A2 (PLA2). Future research should adopt frameworks such as “gut microbiota-SCFAs/BAs-5-HT pathway and associated upstream/downstream molecules” to comprehensively elucidate the mechanisms of TCM in constipation treatment.

5-hydroxytryptamine exhibits complex regulatory roles in constipation pathogenesis, and clarifying its paradoxical expression patterns (e.g., both increased and decreased levels in different contexts) and TCM’s specific regulatory mechanisms represents a key research priority. Notably, female STC patients with low SERT and high 5-HT levels may exhibit reduced colonic smooth muscle responsiveness to acetylcholine (ACh) and 5-HT due to overexpression of progesterone receptors (84, 85). This finding may reconcile conflicting reports of elevated 5-HT in both diarrheal and constipated patients, though it does not exclude potential abnormalities in other neuropeptides in constipated individuals.

Clinical studies frequently lack adequate control groups using 5-HT-modulating positive drugs, and some observational groups combine TCM with western medicines, complicating the assessment of TCM’s independent effects on the 5-HT system (57). Future research should prioritize comparative studies with 5-HT-targeting positive drugs, incorporate measurements of molecules within the “gut microbiota-5-HT pathway-intestinal motility axis,” and deepen investigations into TCM’s therapeutic mechanisms to provide a scientific basis for novel drug development.

## Conclusion

Both clinical and animal studies have largely overlooked constipation accompanied by emotional disorders. Psychological disturbances occur 14 times more frequently in patients with chronic constipation than in the general population, and up to 65% experience some degree of psychiatric symptoms. Given the bidirectional interactions among mental health, the 5-HT system, and gut microbiota, future research should also consider other mechanisms underlying gut–brain axis disruption, particularly low-grade inflammation. Impaired intestinal barrier function and reduced microbial production of short-chain fatty acids can trigger low-grade inflammation, disrupt enterochromaffin cell activity, and alter 5-HT synthesis, ultimately promoting a vicious cycle of psychiatric symptoms and gastrointestinal dysfunction. Investigating how TCM therapies–such as herbal formulas and acupuncture–modulate inflammation-related pathways and restore microbiota–EC–5-HT homeostasis may provide important insights into their regulatory effects on gastrointestinal motility and offer promising directions for future constipation management.
